# Self-Assembly of Metal Nanoclusters for Aggregation-Induced Emission

**DOI:** 10.3390/ijms20081891

**Published:** 2019-04-17

**Authors:** Jianxing Wang, Xiangfang Lin, Tong Shu, Lei Su, Feng Liang, Xueji Zhang

**Affiliations:** 1 Beijing Advanced Innovation Center of Materials Genome Engineering, Research Center for Bioengineering and Sensing Technology, School of Chemistry and Biological Engineering, University of Science and Technology Beijing, Beijing 100083, China; wangjianxing_ustb@163.com (J.W.); 18265166830@163.com (X.L.); shutong@ustb.edu.cn (T.S.); 2 Beijing Advanced Innovation Center for Food Nutrition and Human Health, Beijing Technology and Business University, Beijing 100048, China; 3 The State Key Laboratory for Refractories and Metallurgy, Institute of Advanced Materials and Nanotechnology, School of Chemistry and Chemical Engineering, Wuhan University of Science and Technology, Wuhan 430081, China

**Keywords:** aggregation-induced emission (AIE), metal nanoclusters (NCs), gold nanoclusters, self-assembly of metal nanoclusters

## Abstract

Aggregation-induced emission (AIE) is an intriguing strategy to enhance the luminescence of metal nanoclusters (NCs). However, the morphologies of aggregated NCs are often irregular and inhomogeneous, leading to instability and poor color purity of the aggregations, which greatly limit their further potential in optical applications. Inspired by self-assembly techniques, manipulating metal NCs into well-defined architectures has achieved success. The self-assembled metal NCs often exhibit enhancing emission stability and intensity compared to the individually or randomly aggregated ones. Meanwhile, the emission color of metal NCs becomes tunable. In this review, we summarize the synthetic strategies involved in self-assembly of metal NCs for the first time. For each synthetic strategy, we describe the self-assembly mechanisms involved and the dependence of optical properties on the self-assembly. Finally, we outline the current challenges to and perspectives on the development of this area.

## 1. Introduction

Metal nanoclusters (NCs) consist of several to hundreds of metal atoms, bridging the gap between small organometallic complexes and large metal nanoparticles (NPs). The metal NCs typically have a core–shell structure, which is composed of a metal core and a protective ligand shell. Owing to their ultra-small size (<2 nm), which is comparable to the Fermi wavelength of electrons, the spatial confinement of free electrons in metal NCs leads to discrete electronic transitions, thereby exhibiting intriguing molecular-like properties such as molecular chirality, HOMO–LUMO transitions, and photoluminescence [1,2,3]. However, the quantum yields (QY) of metal NCs seldom exceed 0.1% [4,5], which greatly restrict them in many optical applications, such as biosensing, bioimaging, and solid-state lighting and display [6,7,8,9,10,11]. Recently, a strategy of aggregation-induced emission (AIE) [12] to obtain high luminescence of metal NCs has attracted increasing research interest [13]. The AIE origin of metal NCs could be attributed to the restriction of intramolecular vibration and rotation of the ligand’s shell on the NCs’ surface after aggregation, thereby facilitating the radiative energy transfer via restraining ligand-related nonradiative excited state relaxation [14,15]. So far, the common AIE approaches for metal NCs are cation- and solvent-induced aggregations [14,16,17]. However, both of these two AIE approaches often have the problems of structural irregularity and inhomogeneity due to the random aggregation route, which usually lead to the instability and poor color purity of NC aggregation [14], thereby restricting their potentially practical applications. Therefore, new approaches to synthesize a more regular or homogeneous morphology of NC aggregations are desperately needed.

Self-assembly, as an effective strategy to manipulate the spatial arrangement of nanosized building blocks to form specific structures [18], is considered to be capable of guiding the metal NCs to form a well-defined architecture. Although big success has been achieved in self-assembly of large building blocks, such as metal nanoparticles [19], proteins [20], and polymers [21], it is more difficult to direct metal NCs assemble into high-ordered structures due to their ultra-small size and unique core–shell structure. As to NCs, the large surface energy [22] makes them unstable in self-assembly, thereby resulting in recrystallization or fusing into big nanoparticles. In particular, the interactions between metal NCs originating from the ligand shell on the NC surface are rather weak, comparable to the thermal fluctuation energy of the surroundings, which often leads to the detachment of assembled NCs and formation of irregular structures [23]. Therefore, strengthening the inter-NC interaction through manipulating the outer layer of metal NCs, capping ligands, is critical to the success of self-assembly of metal NCs. So far, directing the capping ligands’ configuration has been applied to the self-assembly of metal NCs mainly in two aspects. The first is to choose appropriate molecules as the capping ligands of metal NCs to direct the spontaneous association of NCs under equilibrium conditions into well-defined assemblies joined by covalent or noncovalent bonds, named “capping ligand induced assembly.” The second is to utilize soft templates to guide the shape-controlled synthesis of metal NCs, named “soft template directed assembly.” In this route, the NC assemblies formed should achieve the shape of the templates. Additionally, limited success has been achieved in utilizing the traditional AIE approaches in self-assembly of metal NCs into highly ordered architecture, including cation- and solvent-induced assembly.

In this review, we first summarize the synthesis strategies developed for the self-assembly of metal NCs into well-defined architectures and describe their optical properties. While there are a few reports of alloy NCs, this review mainly investigates the self-assembly of Au NCs, Ag NCs, and Cu NCs from the standpoint of directing the capping ligand configuration, including capping ligands-, soft templates-, and cation- and solvent-directed assembly. In specific self-assembly strategies, we introduce the driving forces involved in the NCs self-assembly, experimental variables controlling their assembled morphologies, and the dependence of NC optical properties on self-assembled structure. Finally, we outline the current challenges of NC self-assembly and our perspective on the development of this area.

## 2. The Self-Assembly of Au NCs

So far, Au NCs have become the most investigated metal NCs in self-assembling into well-defined architectures. The common strategy is to choose appropriate soft templates to form self-assembled Au NCs in situ, such as amphiphilic hydrocarbon, Au(I)-thiolate complexes, cetyltrimethylammonium bromide (CTAB)-metal halide complexes, polymer, and protein fibrils. A few polymers and proteins could also serve as capping ligands to synthesize Au NCs and endow them with self-assembled behaviors. In addition, several cations have been proven to be capable of triggering Au NC assembly into well-defined structures by electrostatic interactions. Therefore, we introduce the self-assembly of Au NCs on the basis of the three aforementioned synthesis strategies.

### 2.1. Soft Template Directed Assembly

#### 2.1.1. Amphiphilic Hydrocarbon

**Figure 1:**
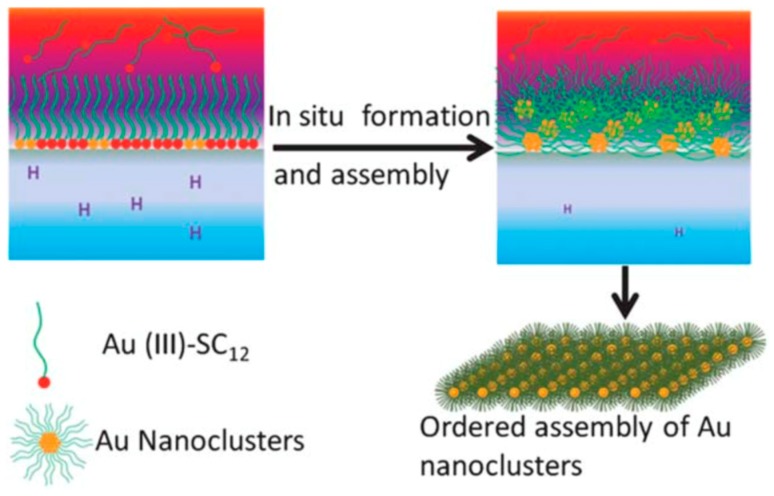
Schematic representation of the in situ formation and the assembly of Au nanoclusters (NCs) at an oil/water interface. Adapted with permission from Ref. [24]. Copyright (2011) Royal Society of Chemistry.

The self-assembly behaviors of hydrocarbon amphiphiles have been applied to Au NCs in two ways. One is to serve as soft templates at a liquid/liquid interface for in situ synthesis of shape-controlled assembled Au NCs. It is well-known that utilizing this interfacial organization technique could successfully guide the self-assembly of colloidal nanoparticles into a well-defined structure, which could also be applicable to the self-assembly of NCs. Utilizing the amphiphilicity of Au NCs precursor, Au (III)-SC_12_, Wang et al. demonstrated the successful self-assembly of Au NCs at the oil/water interface into ordered nanoribbons [24]. Au (III)-SC_12_ has a hydrophilic head of an Au ion and a hydrophobic tail of alkyl, which guide the spontaneous self-assembly at the oil/water interface with Au^3+^ pointing to the water phase. The water phase was added with a reducing agent, which further guaranteed the heterogeneous reduction of Au(III) to Au(0) at an oil/ water interface, leading to in situ formation of self-assembled Au NC nanoribbons at the interface (Figure 1). The as-synthesized Au NC nanoribbons exhibited a large Stokes′ shift and more enhanced emission intensity than randomly dispersed Au NCs. Additionally, self-assembled nanosheets of hydrophobic alkyl thiol-capped Au NCs a single NC thick were also successfully obtained by the liquid/liquid assembly technique. Zhang and Lu investigated the two-dimensional (2D) self-assembly of 1-dodecanethiol (DT)-capped Au NCs in a colloidal solution of two miscible high boiling solvents with a slight difference of polarity [23]. The solvent microphase separation leads to a lamellar interface, which serves as a soft template to guide Au NC self-assembly into single-NC-thick sheets due to an inter-NC isotropic hydrophobic attraction. The morphology of the Au NC assemblies could be further adjusted by the Au NC concentration and solvent volume ratio. Furthermore, Zhang and coworkers reported the 2D self-assembly of DT-capped Au NCs into well-defined sheets with controlled thickness in the colloidal solution [25]. The initial assembly was 1D-oriented, triggered by the anisotropic dipolar attraction between NCs, leading to a redistribution of DT ligands and thereby generating the asymmetric van der Waals attraction between DT. The coordination of these two attractions together allows manipulation of the morphology and thickness of the 2D self-assembly of Au NCs.

On the other hand, amphiphilicity could be introduced into hydrophilic Au NCs by a simple surface modifying approach, thereby guiding the self-assembly of NCs at an air/water interface. For example, the anionic surface of the hydrophilic Au NCs could be modified with hydrophobic cations via a phrase-transfer-driven ion-paring reaction. Inspired by this idea, Xie and Lee successfully synthesized amphiphilic Au NCs by patching hydrophilic 6-mercaptohexanoic acid (MHA)-capped Au NCs with hydrophobic cetyltrimethylammonium ion (CTA^+^) to approximately half of a monolayer coverage [26]. Owing to the coexistence of hydrophilic MHA and hydrophobic MHA-CTA^+^ in a comparable ratio, the as-prepared Au NCs exhibited excellent solubility in solvents with different polarities and molecular-like amphiphilicity, and could self-assemble into staked bilayers at the air/liquid interface.

#### 2.1.2. Au(I)-Thiolate Complexes

As one of the common Au(I)-thiolate complexes, Au(I)-cysteine complexes are well known to self-assemble into irregular architectures with sizes larger than 500 nm at an acidic pH [27]. A recent work suggests that the structural irregularity of Au(I)-cysteine complexes is related to the chirality of cysteine [28]. Specifically, the self-assemblies of pure L-cysteine-Au(I) or D-cysteine-Au(I) complexes are disordered and irregular with diameters larger than 500 nm, which is accordant with the aforementioned report. However, using the mixture of L-cysteine and D-cysteine to react with Au(III), the as-prepared L/D-cysteine-Au(I) complexes would self-assemble into a well-defined spindle shape. The morphological changes of Au(I) assemblies are determined to be closely associated with Au(I)-Au(I) aurophilic interactions, and stacked zwitterionic interactions and hydrogen bonding between L/D-cysteine ligands. Moreover, the Au(I)-cystine assemblies could serve as soft templates to prepare highly emissive Au NCs in situ by the NaBH_4_-mediated reduction.

**Figure 2:**
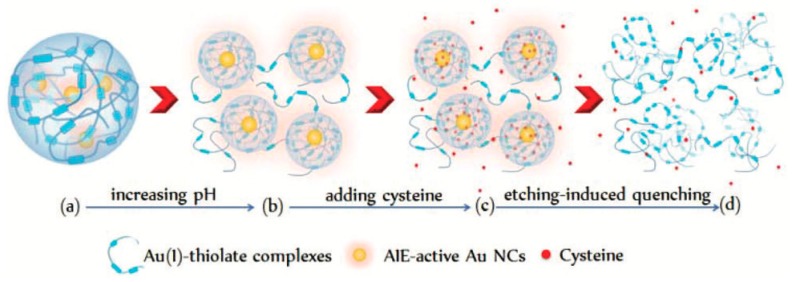
Schematic of the increase in the solution pH, prompting the permeation of the cysteine molecules through the Au(I)–thiolate complex surroundings to etch and quench the embedded Au NCs. (**a → b**) Increasing the pH prompts the disassembly of the Au(I)–thiolate complex network encapsulating the AIE-active Au NCs and enhances the emission; (**b → c**) alkaline pH facilitates the penetration of cysteine through the Au(I)–thiolate surroundings, thereby allowing access to the embedded Au NCs; (**c → d**) cysteine molecules etch the Au(0) cores, causing the decomposition of the AIE-active Au NC system and quenching of the emission. Adapted with permission from Ref. [30]. Copyright (2018) Royal Society of Chemistry.

On the other hand, our group recently found that Au(I)-glutathione (GSH) complexes were capable of forming crystalline networks encapsulating a great many Au NCs into nanoparticles in acidic pH [29]. The crystalline networks of Au(I)-GSH complexes could further disassemble by increasing the solution pH, thereby generating varied aggregation-induced emission (AIE) with a QY as high as 14%. Interestingly, the pH-induced disassembly results in the finding of a certain degree of crystallization occurring on the surface of Au NCs, expanding the knowledge of the surface/interfacial structures of AIE-type Au NCs. Additionally, the disassembled behaviors of the Au(I)-GSH thiolate crystalline networks surrounding Au NCs could further function as a pH-sensitive “valve” to control the access of environmental chemicals to the inner Au(0) core of the NCs. For example, for small molecules, such as cysteine, the Au(I) crystalline networks have pH-sensitive permeability and as the solution pH increased, the disassembly of crystalline networks would facilitate cysteine gaining access to the embedded Au NCs and etching the Au(0) core (Figure 2). Based on this idea, our group recently developed an ultra-sensitive cysteine sensor at alkaline pH, exhibiting an ultra-wide linear concentration range of nine orders of magnitude and an ultra-low limit of detection of 6.3 pM [30].

#### 2.1.3. CTAB-Au Halide Complexes

Utilizing CTAB as a surfactant and thiourea as a reducing agent, Au halide salts have been employed to synthesize mesoscale assemblies of Au NCs with well-defined boundaries. The Au halide complex anions have a stronger affinity than Br^−^ to bind to CTA^+^, leading to weakened electrostatic repulsion between cationic CTA^+^, which facilitates the formation of hierarchical mesoscale micelles. The micelles of CTAB-Au halide complexes further serve as soft templates to guide the preparation and assembly of Au NCs, which have similar morphologies to the ultimate Au NC assemblies [31]. The concentration of CTAB was found to exert great influence on the morphology and packing density of the NC assemblies. For example, the 1D self-assembled nanorods of Au NCs would transform into hollow vesicles as the CTAB concentration increased. The Au NC assemblies templated by CTAB-Au halide complexes have been reported to have excellent performance and reusability in catalytic studies.

#### 2.1.4. Polymer

**Figure 3:**
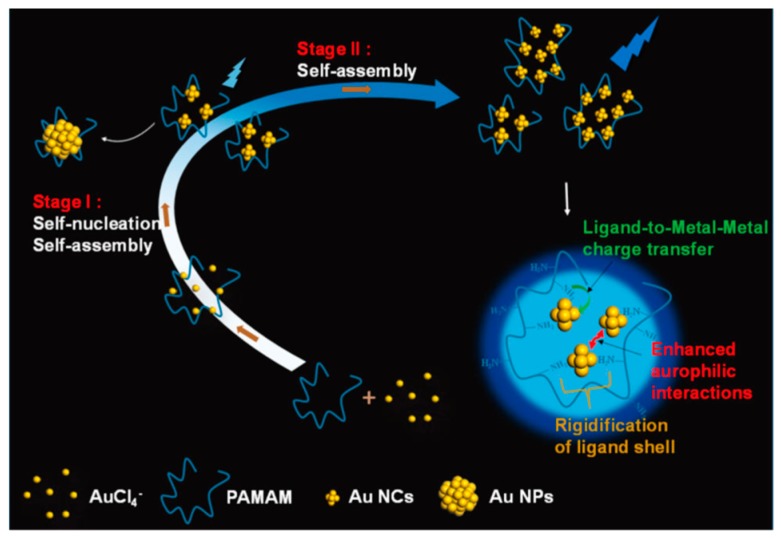
Schematic illustration of growth process and the morphology of Au_5_ self-assemblies in poly(amidoamine) (PAMAM) matrix. Adapted with permission from Ref. [32]. Copyright (2018) American Chemical Society.

Long branching polymers with specific conformations could serve as the backbone for templated synthesis of self-assembled Au NCs. One of the common spherical branching polymers, poly(amidoamine) (PAMAM) dendrimer, has been reported as a soft template to in situ synthesis self-assembled Au NCs. Utilizing the fourth-generation amine-terminated PAMAM dendrimers as capping and hosting ligands, Yang et al. presented a new strategy to successfully prepare highly emissive poly-Au_5_ NCs with a QY of 25% [32]. The formation of poly-Au_5_ NCs involves two stages. The first stage is simultaneous self-nucleation and self-assembly of PAMAM-Au ion complexes into poly-Au_5_ NCs, accompanied with a rapid emission increase. The second stage is a sole self-assembly of these poly-Au_5_ NCs without further reduction, with a relatively slow enhancement of emission but 30% contribution to the emission intensity of the final assemblies (Figure 3). The intensive emission of the as-prepared poly-Au_5_ NC assemblies originates from the more rigid structures reducing the nonradiative excited state relaxation, and the strengthened aurophilic interaction promoting the excited state relaxation dynamics.

In addition, nanohydrogels are capable of serving as soft templates for in situ synthesis of self-assembled Au NCs. The nanohydrogel could be formed by spatially confinement of Au(I)-thiolate complexes in a cationic polymer via electrostatic attraction. For example, Xie and coworkers proposed a simple and rapid in situ synthesis of self-assembled GSH-capped Au NCs within chitosan a nanohydrogel [33]. Chitosan is a polycationic polymer with many positively charged amine groups, the pk_a_ of which is 6.3–6.7, while the GSH ligands of the Au(I)-SG complex have two carboxylic groups (pK_a1_ = 2.12 and pK_a2_ = 3.53). At the gelation pH between the pk_a_ of chitosan and GSH, the electrostatic repulsion between negative carboxylic groups in Au(I)-SG complexes would be greatly weakened through the addition of cationic chitosan, leading to a spontaneous self-assembly of Au(I)-SG complexes into monodispersed nanoparticles. The spatial confinements of Au(I)-SG complexes within the chitosan matrix via electrostatic self-assembly facilitate a more rapid formation of Au NCs than free complexes without confinements. The as-prepared self-assembled Au NCs impregnated in the chitosan nanogel also exhibited a strong emission, due to the inhibition of the nonradiative decay pathway.

#### 2.1.5. Protein Fibrils

The self-assembly of protein-capped Au NCs could be obtained by employing protein fibrils as soft templates to in situ synthesize NCs. It is known that many proteins are able to form well-ordered fibrillar cross-β-sheet structures, which are typical assemblies held together via weak noncovalent forces. For instance, Garcia et al. reported the fibrillation of human-insulin-assisted in situ synthesis of assembled Au NCs [34]. During the fibrillation process of insulin in an alkaline environment, the Au precursor was added under physiological temperature and vigorous stirring and Au NCs were gradually formed by the reduction of insulin. Chattopadhyay and coworkers also proposed the employment of bovine serum albumin (BSA) fibrils as the scaffold for the preparation of self-assembled Au NCs [35]. BSA has a free unpaired cysteine at the 34th position, which helps with dimerization and subsequent well-defined self-assembly, thereby forming BSA fibrils. Using BSA fibrils as stabilizers, the self-assembled Au NCs exhibited enhanced fluorescence and a large red shift of emission, in comparison to their individual counterparts.

### 2.2. Cation Induced Assembly

As a common cationic surfactant, cetyltrimethylammonium bromide (CTAB) can easily bind to nanoparticles with negative charges, such as silica, metal oxides, and quantum dots, due to the coexistence of electrostatic and hydrophobic interactions. As to Au NCs with negative charge, CTAB can build an electrostatic inter-NC connection, thereby guiding their self-assembly in an aqueous solution. Based on this idea, Wu and coworkers recently reported CTAB-induced assembly of GSH-capped Au NCs, which is favored by the electrostatic binding of CTA^+^ to negatively charged carboxyl groups in GSH [36].

Additionally, Zn^2+^ has been shown to function as an external metal ion triggering the self-assembly-mediated emission color tunability of Au NCs. For instance, individual 3-mercaptopropionic acid (3-MPA)-capped Au NCs are non-luminescent. By the addition of Zn^2+^, Au NCs self-assemble randomly via the coordination of Zn^2+^ with carboxyl groups in MPA ligands, and emit intense yellow emissions owing to the restriction of the MPA ligands′ vibrations and rotations. After aging for 24 h, the irregular self-assemblies transform into well-defined one-dimensional (1D) architecture with green emissions, with a QY of 20%. Compared to random assembly, this blue shift in emission is attributed to the ascendancy of inter-aurophilic and Zn^2+^-NC interactions over the intra modes [37]. In addition, Kuppan further found that the highly ordered green emissive Au NC assemblies exhibited much better emission anisotropy than random assemblies, revealing that the directionality in self-assembly of Au NCs makes a great contribution to the emission polarization [38].

### 2.3. Ligand Induced Assembly

#### 2.3.1. Polymer Micelles

Polymer micelles are one of the most common drug delivery nanosystems for anticancer therapy, exhibiting excellent biocompatibility and pharmacokinetic control. As the representative of polymer micelles, self-assembled diblock copolymers consisting of a thermosensitive poly(N-isopropylacrylamide) (PNIPAm) and a hydrophilic poly(ethylene glycol) (PEG) block have been proven to be a simple and useful platform for drug delivery. The PNIPAm has a cloud point temperature of 32 °C, leading to their self-assembly into micelles in the biological environment, which is stablished by the PEG corona to prevent aggregation. Thermosensitive and thiol-terminated PEG-PNIPAm could be further employed as a capping agent to synthesize Au NCs, which would self-assemble into micelles above their lower critical solution temperature, accompanied with an enhanced emission. The as-prepared thermosensitive Au NC-polymer micelles show great potential for fluorescent live cell imaging [39].

#### 2.3.2. Protein

**Figure 4:**
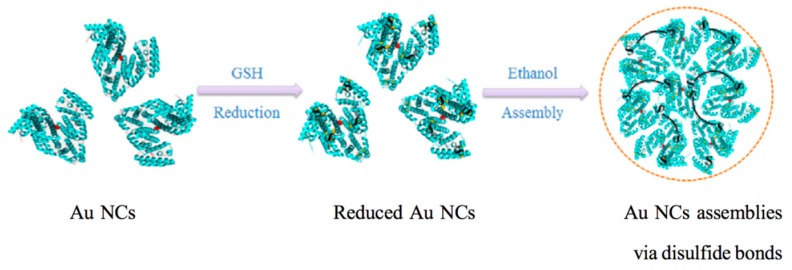
Schematic illustration for the self-assembly of protein-capped Au NCs by a protein cross-linking approach. Adapted with permission from Ref. [40]. Copyright (2017) Elsevier.

Protein-capped Au NCs can self-assemble into larger nanoparticles in the presence of GSH via a protein cross-linking approach. Using GSH as an endogenous reductant, the intramolecular disulfide bonds within the protein ligands on the surface of Au NCs were cleaved. The obtained free -SH groups assembled again through intermolecular disulfide bonds into protein nanoparticles, resulting in cross-linking and self-assembly of the protein-capped Au NCs (Figure 4). Compared to the individual Au NCs, the NC assemblies exhibited good biocompatibility, improved cellular uptake, highly precise tumor targeting, and excellent performance as photosensitizers [40]. Moreover, Shen and Cai successfully encapsulated indocyanine green (ICG) into the as-prepared Au NC assemblies via noncovalent binding for therapeutic real-time monitoring on the basis of fluorescence resonance energy transfer (FRET) [41]. Au NCs-ICG nanoprobes (Au NCs-INPs) also showed excellent dual-modal near-infrared fluorescence and photoacoustic imaging, improved cancer cell killing, and tumor removal efficiency in the simultaneous photodynamic therapy and photothermal therapy.

### 2.4. Effects of Different Self-Assembled Strategies on Au NCs’ Optical Properties

The strategy of soft template-directed assembly usually has a great influence on the luminescence of Au NCs. On one hand, the components of some soft templates can themselves be used to be as capping ligands to in situ synthesize Au NCs, thereby forming a more compact and rigid ligand shell on the surface of the Au(0) core to enhance Au NCs’ luminescence via the AIE mechanism. For instance, Au(I)-cysteine assemblies strongly staple on the surface of inner formed Au(0) cores, which reduces the PL quenching by collision and restrains the intramolecular vibration- and rotation-induced internal nonradiative relaxation pathways, thereby obtaining a high QY of ~10% [27,28]. In our previous work, during the formation of Au NCs within the crystalline Au(I)-GSH networks, some Au(I)-GSH complexes could insert their thiol ligands into the shell of Au NCs to form strong aurophilic interactions with the Au(I) of the staple-like motifs binding to the Au(0) core, leading to the formation of a crystalline and hence more compact shell, thereby contributing to the QY of ~ 14% [29]. On the other hand, some templates could only function as the supporting matrix to confine Au NCs in limited space, which resulted in the matrix-coordinate-induced aggregation restraining the nonradiative relaxation channels via locking the ligands of Au NCs in the matrix, thereby generating an intense AIE effect. For instance, GSH-capped Au NCs impregnated within the confined space in chitosan nanogel have a strong coordination between -COOH of GSH with negative charges, and -NH_2_ of chitosan with positive charges. This intense coordination could further restrict the GSH ligands’ intra- or intermolecular vibration- and rotation-induced nonradiative relaxation pathways, thereby contributing to the luminescent intensity to a major extent [33].

In addition, the cation-induced assembled strategy, which could crosslink Au NCs in an ordered aggregated route, is more like a special case of the conventional cation-induced AIE method. The coordination of cations with the negatively charged groups in capping ligands on NC surfaces rigidifies the surface ligand shell, thereby restraining the intramolecular motion (RIM) to generate intense AIE [36,37,38]. Additionally, for the strategy of ligand-induced assembly, when the morphology of the ligand shell of Au NCs undergoes deformation during the ligand-induced self-assembly process, the PL intensity of the Au NCs is more likely to be enhanced via AIE mechanism. For instance, BSA-capped Au NCs can self-assemble via a protein cross-linking approach with negligible change in optical properties compared to the individual NCs, because the BSA shell is relatively rigid and its intramolecular rotations and vibrations are unaffected during assembly [40,41]. Contrarily, self-assembled PNIPAm-capped Au NCs exhibited enhanced luminescent intensity above the clouding temperature, owing to the formation of more compact and rigid PNIPAm self-assembled structures around Au NCs, thereby inducing the RIM effect to generate strong AIE [39].

## 3. The Self-Assembly of Ag NCs

Self-assembly studies of Ag NCs are still in the preliminary stages, and have achieved limited success in strategies of capping ligand- and solvent-induced assembly into well-defined structures. However, DNA-directed self-assembly of Ag NCs is being intensively investigated because DNA could not only serve as capping ligands for Ag NCs, but also as versatile building blocks for programmable assembly. Therefore, we will describe the self-assembly of Ag NCs in three ways: DNA-, capping-ligand-, and solvent-induced assembly.

### 3.1. DNA-Induced Assembly

DNA-capped Ag NCs have attracted tremendous attention as a novel powerful fluorescence nanomaterial, which are well-known to exhibit sequence-dependent emission. Some efforts have been made to spatially manipulate the self-assembly of Ag NCs through DNA nanostructures. By utilizing sequence-specific loops as the stabilized ligands, Orbach et al. proposed two approaches to synthesis of self-assembled nucleic-acid-capped Au NC nanowires with red or yellow emission [42]. One is through the hybridization–polymerization process of nucleic acids. The other is through the nucleic-acid-driven hybridization chain reaction. Additionally, Ye et al. presented another simple method to assemble Ag NCs and a G-rich strand into nanowires in the presence of a long, enzymatically produced scaffold [43]. The scaffold drives a number of Ag NCs and G-rich strands in proximity, which leads to an approaching of the end of G-rich strands to NCs, thereby generating a great enhancement in emission.

**Figure 5:**
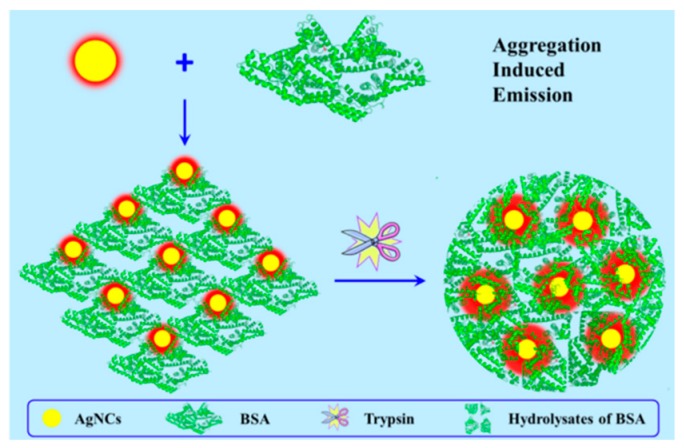
Schematic representation of the response self-assembly of dsDNA-Ag NCs to bovine serum albumin (BSA) and trypsin. Adapted with permission from Ref. [44]. Copyright (2017) Elsevier.

On the other hand, the double-stranded-DNA-stabilized Ag NCs (dsDNA-Ag NCs) could self-assemble into a large sheet-like membrane in solution driven by bovine serum albumin (BSA), thereby generating AIE-induced five-fold emission enhancement and a blue shift in emission [44]. After addition of digestive enzyme, the irregular morphology of Ag NC assemblies would transform into large, well-defined particles with more significant emission enhancement (30-fold), owing to their altered surface (Figure 5). In addition, Wu and coworkers recently reported two other self-assembled modes of dsDNA-Ag NCs [45]. Through the co-assembly of dsDNA-Ag NCs and human papillomavirus (HPV), 16 main capsid protein L1, empty HPV virus-like particles (VLPs) were formed in assembly buffer, of which the cavities were further bound with dsDNA-Ag NCs, exhibiting enhanced emission, while the post-assembly induced the binding of dsDNA-Ag NCs to the external surface of VLPs, which showed no enhancement. Accordingly, the co-assembly of capsid and dsDNA-Ag NCs provide a novel emissive method to monitor the process of in situ VLP self-assembly.

### 3.2. Solvent-Induced Assembly

**Figure 6:**
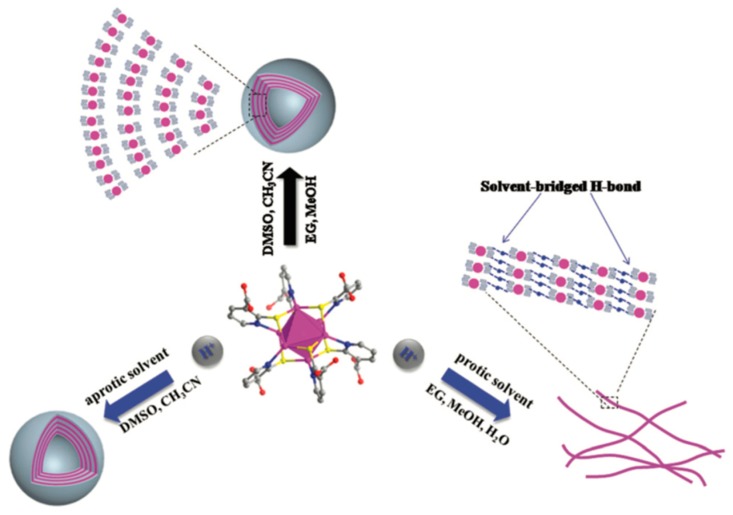
Schematic representation of the building block of Ag_6_-NC for the morphological evolution process controlled by molecular structure and solvents into vesicles and nanowires. Adapted with permission from Ref. [46]. Copyright (2017) Royal Society of Chemistry.

Manipulating the self-assembly of Ag NCs based on non-covalent forces through modulating the interactions between building blocks and solvents. Shen et al. reported the controlled assembly of mercaptonicotinate (MNA)-capped Ag NCs into multilayer vesicles or nanowires in different solvents [46]. After protonating the Ag NCs by adding hydrochloric acid, the morphology of self-assembled vesicles kept stable in aprotic solvents, such as DMSO and CH_3_CN, while in protic solvents, such as water, MeOH, and EG, the vesicular morphology would transform into nanowires (Figure 6). The formation of self-assembled nanowires originates from the strong solvent-bridged hydrogen bonding and the π–π stacking interactions between MNA. Furthermore, the obtained nanowires of Ag NCs could self-assemble into hydrogels, which have a high water content of 99.5% and excellent self-healing and mechanical strength properties. Additionally, Gao et al. recently reported another example of solvent-induced self-assembly of Ag NCs [47]. Using water-soluble N-acetyl-L-cysteine (NALC) as reducing and capping ligands, atomically precise Ag_6_ NCs were synthesized. By introducing ethanol to the aqueous solution of NCs, the NALC-capped Ag_6_ NCs further self-assembled into ultrafine nanowires, long ribbons, and 3D porous networks, owing to the solvent polarity, van der Waals, and electrostatic interactions between NALC ligands. Such self-assembly of Ag NCs exhibits a great potential in the future manufacture of nanodevices based on Ag NCs.

### 3.3. Ligand-Induced Assembly

**Figure 7:**
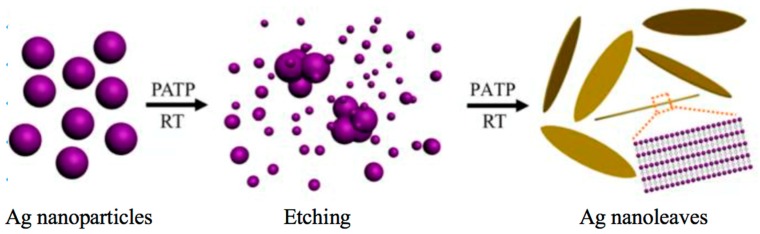
Schematic illustration for the process of etching induced formation of assembled lamellar Ag nanoleaves composed of Ag NCs and p-aminothiophenol (PATP). Adapted with permission from Ref. [48]. Copyright (2013) American Chemical Society.

Self-assembled Ag NCs can be obtained from a bottom-up route by choosing appropriate capping ligands. Li et al. developed a ligand etching strategy to direct the self-assembly of NCs into lamellar supramolecular structures [48]. By employing p-aminothiophenol (PATP) as an etchant, self-assembled lamellar Ag nanoleaves composed of Ag_25_ NCs and PATP spontaneously formed from etching 4 nm Ag nanoparticles (Figure 7). The mechanism of assembly was revealed as a two-step reaction. First, the 4 nm Ag nanoparticles were rapidly etched by PATP into ~1 nm Ag_25_ NCs, which were further interconnected by PATP to form Ag_25_-PATP-Ag_25_ complexes owing to the electrostatic and covalent interactions. Second, these Ag_25_-PATP-Ag_25_ complexes served as building blocks to assemble lamellar Ag nanoleaves, due to the intense dipole–dipole interaction and π–π stacking force between the neighboring rigid benzene skeleton of PATP. Although this strategy shows a vital route to design novel morphologies of Ag assemblies, the two-step assembly process is complicated and time-consuming. Jia et al. reported a more effective and straightforward avenue to bottom-up synthesis of self-assembled Ag NCs [49]. Utilizing D-penicillamine (DPA) as a reducing and capping agent, the self-assembly of Ag NCs could be obtained by one-pot microwave-assisted synthesis. Through tuning the synthesis conditions, including the precursor concentration, chirality of DPA, environment temperature, and limited reaction volume, the Ag NCs would self-assemble into different morphologies with varied emission colors. The as-prepared Ag NC assemblies also exhibited intense emissions with a QY as high as 25.6%, owing to the mechanism of AIE. In addition, one of the morphologies of NC assemblies, the lamellar supramolecular structure, possesses excellent electrical conductivity due to its well-confined and closely packed architecture.

### 3.4. Effect of Different Self-Assembly Strategies on Ag NCs’ Optical Properties

The DNA-induced assembly strategy has an important influence on the luminescence of DNA-capped Ag NCs. On one hand, the luminescent intensity of self-assembled DNA-Ag NCs nanowires could be enhanced though “G-rich” sequences [43]. It is well-known that the proximity of “G-rich” sequences to the Ag NCs would separate Ag NCs from the solvent to form a better protected environment around NCs, thereby reducing the nonradiative relaxation pathways to obtain a luminescent enhancement [50]. On the other hand, the introduction of capsids or protein to DNA-capped Ag NCs could result in the self-assembly process of Ag NCs through capsid- or protein-induced DNA assembly. Compared to the individual Ag NCs, the luminescent intensity of the self-assembled Ag NCs would be enhanced owing to the RIM effect, thereby generating strong AIE [44,45].

Additionally, some specific capping ligands are able to endow Ag NCs with the capability to self-assemble into well-defined structures, accompanied with enhanced luminescent intensity and tunable emission color. The luminescence of Ag NC assemblies originates from LMCT or LMMCT and subsequent radiative relaxation via triplet excited states. Compared with individual Ag NCs, the self-assembled NCs usually have stronger hydrogen bonding and Ag(I)···Ag(I) interaction, which restricts the intramolecular motions to reduce the energy loss from the nonradiative pathways, thereby generating enhanced luminescence. Moreover, multicolor emissive Ag NC assemblies can be obtained by adjusting synthetic conditions, for example, the ligand chirality or the precursor concentration. The tunable emission color of NC assemblies is related to their varied distance of the Ag···Ag interaction through the adjustment of the hydrogen bonding and Ag···Ag interaction within assemblies [49].

## 4. The Self-Assembly of Cu NCs

In comparison to the noble metals Au and Ag, metal Cu is widely used in industry due to its abundant reserves, relatively low price, and high conductivity. As the size of metal Cu is confined to below 2 nm, Cu NCs exhibit unique photoluminescence (PL) and enhanced electrocatalytic performance compared with their larger nanometer-sized and bulk counterparts. However, the PL intensity of individual Cu NCs is normally very weak and the emission color is hard to control, owing to the weak restriction of the ligands′ vibrations and rotations. Additionally, individual Cu NCs can be easily oxidized and aggregate both in storage and further employment, which greatly weakens their stability in practical applications.

In contrast to individual Cu NCs, self-assembled Cu NCs exhibit enhanced PL intensity, broad emission color tenability, and excellent stability, showing tremendous potential in many fields, especially biosensing, bioimaging, LED, and electrocatalysis in oxygen reduction reactions (ORR). However, the self-assembly of ultra-small Cu NCs is still challenging and has achieved limited success. Next, we will introduce the self-assembly strategies of Cu NCs from the standpoint of capping-ligand-, hydrogel- and cation-induced assembly.

### 4.1. Hydrogel-Templated Assembly

Hydrogel has been recently employed as the soft template to direct shaped-controlled synthesis self-assembled Cu NCs. Rogach and coworkers reported the in situ preparation of composite films incorporating Cu NCs, wherein the NCs were impregnated into a 3D hydrogel network of polyvinylpyrrolidone (PVP) and poly(vinyl alcohol) (PVA) [51]. This strategy can allow us to produce large area films of Cu NCs and avoid the use of toxic organic solvents and heavy metal elements. The as-synthesized Cu NC hydrogel film exhibited strong orange emissions with a QY of 30% through the strengthened LMCT, followed by a radiative relaxation pathway after hydrogel dehydration.

### 4.2. Cation-Induced Assembly

Very recently, Li et al. proposed a metal ion (Ce^3+^)-induced self-assembled strategy to rearrange the morphology of irregular aggregated cysteine-capped Cu NCs through a crosslinking pathway, leading to the formation of well-defined mesoporous self-assembled spheres [52]. Cysteine-capped Cu NCs are well-known to form aggregates in an acidic environment, emitting strong emissions due to the AIE mechanism. However, their morphologies are quite irregular, owing to a random connection with each other in a pretty fast route. To slow down the velocity of aggregation, a two-step reaction was presented as follows. First, a relatively weak alkaline Na_2_CO_3_ was employed to disperse the irregular aggregates of cysteine gently by releasing OH^−^ via a hydrolysis route. Second, Ce^3+^ was added to neutralize the hydrolyzed OH^−^ from Na_2_CO_3_, leading to an increase of pH to the neutral value, and crosslinking the dispersed NCs to assemble again in an ordered way. The as-prepared Cu NC assemblies exhibited better performance in stability and color purity tests than the irregularly aggregated NCs.

### 4.3. Ligand-Induced Assembly

Capping-ligand-directed assembly is the most common strategy for synthesizing Cu NCs. Besides acting as reducing and stabilizing agents, the capping ligands on the metal core surface have been known to exert a great influence on the PL of metal NCs, via charge transfer from the ligands to the metal atom core (e.g., LMCT and LMMCT) or direct donation of delocalized electrons from electron-rich groups or atoms in the ligands to the metal core. In this respect, the capping ligands of self-assembled Cu NCs not only function as building blocks to construct different morphologies, but also play an important role in enhancing the PL intensity and broadening emission color tunability of the NC assemblies. So far, great efforts have been paid to studying the ligand engineering in self-assembly of Cu NCs to improve their stability and emission, such as utilizing stiffening ligands and ligands with electron-rich groups or atoms. Accordingly, we will describe the capping-ligand-directed Cu NC self-assembly strategy based on different types of stabilizers.

#### 4.3.1. Alkyl Thiols

The alkyl thiols are conventionally adopted stabilizers in the synthesis of metal NCs due to their strong interaction with metals. 1-dodecanethiol (DT), as the most commonly used alkyl thiol capping ligand in self-assembly of Cu NCs, was shown to be an excellent stabilizer for the synthesis of Cu NCs by reducing Cu^2+^ in dibenzyl ether (BE). The directly synthesized DT-capped Cu NCs in BE were individual and showed no visible emission by 365 nm excitation. After annealing treatments to facilitate the dynamic mobility of DT ligands, these individual Cu NCs with poor emission could further self-assemble into two-dimensional architectures, orientated by the polar attraction between NCs and reinforced by the van der Waals force attraction between DT. By adjusting the annealing temperature, Zhang and coworkers further prepared self-assembled 2D ribbons of DT-Cu NCs in BE with varied compactness [53]. More compacted assemblies of NCs emit stronger emissions, owing to the strengthened inter- and intra- coprophilic interactions and weakened intramolecular vibration and rotation of DT ligands. Meanwhile, the improved compactness introduces additional Cu(I)-Cu(I) cuprophilic interactions of inter-NCs leading to a blue shift in emission, leading to tunable emission color from yellow to blue-green. Self-assembled DT-capped NCs with different emission colors were employed to fabricate NC-based white LEDs.

**Figure 8:**
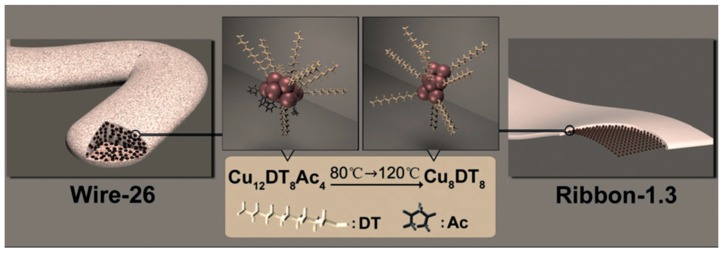
Evolution of Cu NC self-assembly architectures from wire to ribbon. Adapted with permission from Ref. [54]. Copyright (2014) Wiley.

Self-assembled ribbons of DT-stablished Cu NCs could also be obtained via direct synthesis of Cu NCs in a mixed solvent of BE and liquid paraffin (LP), using DT as reducing agent. The spontaneous self-assembly of Cu NCs in the colloidal solution was controlled by dipole-induced asymmetric van der Waals attraction. By tuning these two driven forces via annealing treatments, the thickness of the self-assembled ribbons could be adjusted to one single NC scale at high annealing temperatures (Figure 8). Due to the strong van der Waals force inter-NCs, the self-assembled ribbons were free-standing and could be collected as electrocatalysts for ORR. Compared to the individual Cu NCs, the ribbons exhibited improved stability and excellent electrocatalytic capability [54].

In addition, many efforts have been made to further control the self-assembly of the DT-protected NCs. First, light-controlled self-assembly of DT-capped Cu NCs. Zhang′ s group modified DT with photo-responsive azobenzene (Azo) group, and used the Azo-DT as capping ligands to synthesize Cu NCs in a colloidal solution of BE and LP [55]. The synthesized NCs subsequently self-assembled into ribbons by dipole-induced asymmetric van der Waals attraction, and could further transform into spheres in response to the irradiation of UV light. The self-assembled ribbons and spheres of Cu NCs were employed in ORR and the ribbons exhibited better catalytic activity. Second, chloride ions oriented self-assembly of DT-capped Cu NCs [56]. Owing to the selective adsorption of chloride ions on the specific facets of Cu NCs, the inter-NC dipolar attraction was weakened, resulting in the redistribution of the DT ligands. Accordingly, the morphology of the self-assembled Cu NCs transformed from 1D nanowires to 2D nanoribbons and nanosheets with increasing concentration of chloride.

Moreover, the metal defects in NC assemblies have been reported to exert a great influence on the emission intensity and color turnability of self-assembled Cu NCs. The contribution of metal defects in NC self-assembly is revealed by accelerating the self-assembly process to deliberately create more metal defects on the surface using ethanol. The metal-defect-rich nanosheets have been determined to possess a high percentage of Cu(I), which facilitates the radiative relaxation pathways by ligand-to-metal-metal charge transfer (LMMCT). Accordingly, the quantum yield (QY) of NC assemblies is greatly enhanced (15.4%), and the emission of the NC assemblies is red-shifted [57]. Inspired by the contribution of Cu(I) metal defects to emission, Au(I) metal defects were doped into the self-assembled nanosheet of Cu NCs to form an additional Au(I)-centered state [58]. The doped Au(I) metal defects introduced new Au(I)-Cu(I) metallophilic interactions, which resulted in the ligand-to-Cu-Au charge transfer facilitating the radiative relaxation pathway, thereby enhancing the emission intensity. Meanwhile, the Au(I) defect doping lowered the energy, leading to a red shift of emission. Only 0.3% of Au(I) doped in assemblies could induce a four-fold emission enhancement and a 100 nm red shift of the emission. The mixture of self-assemblies of NCs with different emission colors and high emission intensity was employed as excellent phosphor in white LED.

#### 4.3.2. Aromatic Thiol

Compared to alkyl thiols, the aromatic thiols possess conjugated benzenes, the electronic structures of which are able to be flexibly controlled by altering substitutional groups. The NCs stablished by the aromatic thiols usually exhibit unique electronic structures, electrochemical properties, and surface chemistry. Moreover, aromatic thiol stabilizers increase the electron delocalization of NCs, leading to red shift of the emissions. On the basis of the advantages of the aromatic-thiol-capped Cu NCs, replacing the conventional alkyl thiols with aromatic ones as reducing and capping agents to synthesize self-assembled Cu NCs has a great effect on LMCT and LMMCT, resulting in emission enhancement and emission color tunability. For example, utilizing 2,3,5,6-tetrafluorothiophenol (TFTP) as a reducing and capping agent, self-assembled nanoribbons of Cu NCs were easily synthesized, for which the absolute quantum yield (QY) was as high as 43.0% [59]. Additionally, by using different aromatic thiols with varied conjugation capabilities as capping ligands, the emission color of NC assemblies can be tuned from yellow to dark red and their QY can reach as high as 15.6% [60]. Moreover, the aromatic-thiol-capped self-assembled Cu NCs could be also obtained by a bottom-up synthesis through a ligand exchange reaction of individual Cu NCs with aromatic-thiols and a spontaneous self-assembly. For example, the self-assembled MUA-capped Cu NCs could be prepared by this bottom-up synthetic strategy, exhibiting permanent excimer-like physics and controlled optical properties [61].

The isomeric effects of aromatic thiols as capping ligands on the self-assembly of Cu NCs were further examined. Using three isomers of mercapto-benzoic acid (MBA) as reducing and capping agentd, one-pot synthesis of self-assembled Cu NCs was developed [62]. The assemblies capped by the three isomers of MBA (TA, 3-MBA, 4-MBA) exhibited different optical and physical properties, such as varied emission color, emission intensity, and pH-induced morphologies. More attempts have been made to control the self-assembly process of aromatic-thiol-capped Cu NCs. By adjusting the experimental variables during self-assembly (e.g., temperature, duration of assembly, the solvents…) to influence the weak interaction between NCs, the inter-NC distance in the assemblies can be further controlled, leading to the variation of photophysical properties, especially allowing emission color tunability and controllability of emission intensity via ligand-to-Cu-Cu charge transfer [63].

### 4.4. Effect of Different Self-Assembly Strategies on Cu NCs’ Optical Properties

The ligand-induced assembly strategy plays an important role in self-assembly of Cu NCs. Through control of the compactness of Cu NC assemblies by adjusting the experimental parameters, including the annealing treatment, the concentration of chloride ions, the solvent, etc., enhanced luminescence intensity and tunable emission color of self-assembled Cu NCs can be obtained. High compactness introduced new inter-NC cuprophilic interaction to increase the average distance between adjacent Cu(I) atoms, thereby resulting in the blue emission shift of self-assembled Cu NCs. In addition, the enhanced compactness strengthened the inter-NC cuprophilic interaction, facilitating the excited-state relaxation and restrain the inter- or intramolecular vibrations and rotations of capping ligands reducing the nonradiative pathways, together leading to the enhanced luminescence via AIE mechanism.

In addition, the dehydration process in hydrogel-templated Cu NC assembly exerts an important effect on the PL intensity of Cu NC assemblies through the AIE mechanism [51]. Before dehydration, the Cu NCs impregnated in the hydrogel experience relatively flexible motion of capping ligands on the Cu(0) core, thereby increasing energy loss via nonradiative pathways, and the NC assemblies exhibit weak luminescence. However, after dehydration, the hydrogel becomes more compact and rigid, restricting the RIM of ligands on the NC surface, thereby generating strong AIE. It is also possible that the inter-NC interactions become strengthened, owing to the formation of the additional cuprophilic interaction. Additionally, the luminescence intensity of Cu NCs could be enhanced through cation-induced NC self-assembly into highly ordered structures. For instance, the crosslinking of Cu NCs by Ce^3+^ self-assembles into well-ordered mesoporous spheres, which are compact to induce RIM effect, thereby exhibiting enhanced luminescent intensity via AIE mechanism [52].

## 5. Summary and Future Perspective

In summary, we discovered that the self-assembly technique could be applied to metal NCs by conducting their capping ligands′ configuration to introduce additional noncovalent or covalent forces to the inter-NCs interactions, such as amphiphilicity, electrostatic interactions, van der Waals forces, hydrogen bonding, or disulfide bonds, thereby guiding them to spontaneously organize into well-define and stable architectures. Three fundamental self-assembly strategies of metal NCs are presented, including soft template-, capping-ligand-, solvent-, and cation-induced assembly. Moreover, the self-assembly strategies of every metal NC exhibit different characteristics. As to Au NCs, the most common strategy is utilizing different soft templates to direct the shape-controlled assembly, such as amphiphilic hydrocarbon, Au(I)-thiolate complexes, CTAB−metal halide complexes, polymer, and protein fibrils. As to Ag NCs, DNA-induced assembly is most widely studied, in which DNA not only serves as the capping ligand for Ag NCs, but also functions as a building block to construct well-defined structures. As to Cu NCs, using different types of stabilizers to endow Ag NCs with self-assembly capabilities has been well investigated, including alkyl thiols and aromatic thiols.

In addition, we discovered that the self-assembly process has a great influence on the optical properties of NCs. After self-assembly, metal NCs exhibit enhancing emission intensity, owing to the strengthened inter- and intra-metallophilic interactions and weakened ligand vibrations and rotations. Meanwhile, some of the assembled NCs showed tunable emission color by tuning the inter-NC metallophilic interactions to obtain blue or red shifts in emission. Moreover, these NC assemblies exhibit excellent performance in extensive fields, including biosensing, drug-delivery, bioimaging, light-emitting diodes, and electrocatalysts.

Despite that these results are encouraging, the self-assembly of metal NCs is still in a preliminary stage and needs new breakthroughs. First, many of the synthetic routes of assembled NCs involve the use of organic solvents, which causes severe environmental problems. More attention should be paid to the self-assembly of NCs in aqueous solution. Second, most of the NC assemblies are on the large size, from several hundred nanometers to mesoscale, which greatly restricts their bioimaging and biomedical applications in future. Further studies should make more efforts to synthesize novel architecture in nanoscale exploring greater possibilities in the biological area. Third, although several environmental factors have been discovered to have a great influence on NCs self-assembly process, such as solvent, temperature, the solution pH, and so on, rational control of the self-assembly process of metal NCs is still one of the key challenges. More efforts should be made to develop novel stimulus-responsive assemblies of NCs to control their morphology and optical properties. Therefore, new advances of synthetic routes and construction of intelligent nanoassemblies are still needed for metal NCs to obtain better performance in practical applications.
